# Evaluation of the Size of a Defect in Reinforcing Steel Using Magnetic Flux Leakage (MFL) Measurements

**DOI:** 10.3390/s23125374

**Published:** 2023-06-06

**Authors:** Jamal Yousaf, Regidestyoko Wasistha Harseno, Seong-Hoon Kee, Jurng-Jae Yee

**Affiliations:** 1 Department of ICT Integrated Ocean Smart Cities Engineering, Dong-A University, Busan 49304, Republic of Korea; j.yousaf@donga.ac.kr (J.Y.); regidestyoko@donga.ac.kr (R.W.H.); 2 National Core Research Center for Disaster-Free and Safe Ocean Cities Construction, Dong-A University, Busan 49304, Republic of Korea

**Keywords:** magnetic flux leakage, defect size, reinforcing steels, sensors, permanent magnets

## Abstract

This study aimed to evaluate 2D magnetic flux leakage (MFL) signals (B_x_, B_y_) in D19-size reinforcing steel with several defect conditions. The magnetic flux leakage data were collected from the defected and new specimens using an economically designed test setup incorporating permanent magnets. A two-dimensional finite element model was numerically simulated using COMSOL Multiphysics to validate the experimental tests. Based on the MFL signals (B_x_, B_y_), this study also intended to improve the ability to analyze defect features such as width, depth, and area. Both the numerical and experimental results indicated a high cross-correlation with a median coefficient of 0.920 and a mean coefficient of 0.860. Using signal information to evaluate defect width, the x-component (B_x_) bandwidth was found to increase with increasing defect width and the y-component (B_y_) amplitude rise with increasing depth. In this two-dimensional MFL signal study, both parameters of the two-dimensional defects (width and depth) affected each other and could not be evaluated individually. The defect area was estimated from the overall variation in the signal amplitude of the magnetic flux leakage signals with the x-component (B_x_). The defect areas showed a higher regression coefficient (R^2^ = 0.9079) for the x-component (B_x_) amplitude from the 3-axis sensor signal. It was determined that defect features are positively correlated with sensor signals.

## 1. Introduction

Reinforcing steel, commonly known as “rebar”, is used to strengthen concrete structures. Rebar adds tensile strength to concrete since concrete is weak under tension, while steel is strong. By strengthening a structure with rebar, its tensile strength is significantly increased. Continuous ribs on the surface of the rebar improve its adhesion to concrete and reduce its slipperiness. It is important to detect and quantify embedded reinforcing steel defects in reinforced concrete (RC) structures, such as buildings and bridges, to maintain and repair them. In smart and ocean cities around the world, the detection and quantification of unseen defects in existing reinforced concrete members is a high-priority research task. Reinforcing steel corrosion is a major cause of deterioration in environmentally exposed existing structures in terms of service life and load capacity [1]. In the presence of corrosion, reinforcing steel is reduced in cross section, resulting in irregular distributions of tensile stresses [2,3]. Condition assessment of reinforced concrete structures is important from an infrastructure management agency’s perspective. This is performed to monitor the severity of deterioration in structures and to take appropriate maintenance measures, if necessary, thereby prolonging the service life of the structure [4]. Quantifying defects in reinforcing steel within a concrete structure and estimating defect condition in advance of premature destruction can prevent huge operational costs.

Nondestructive test (NDT) methods are being developed in many countries to evaluate reinforcing steel corrosion in concrete. Among these methods are visual inspection, electrochemical methods such as half-cell potential, electrical resistivity, the galvanic pulse method, polarization resistance, acoustic emission, impact echo, surface wave measurements, ground-penetrating radar (GPR), ultrasonic pulse velocity (UPV), and optical sensing methods [5]. An overview as well as comparison of the different NDT methods for monitoring reinforced concrete corrosion was published by Zaki et al. [5], including their disadvantages, advantages, and corrosion assessment. GPR provides a qualitative analysis of corrosion damage by the electromagnetic wave attenuation caused by corrosion materials and chloride content [6,7,8]. Conversely, infrared thermography detects any abnormal distribution of concrete temperature due to rebar corrosion and cracks inside the concrete [9,10,11]. The corrosion effects on the mechanical properties of concrete are most accurately estimated using elastic wave methods [12]. In order to adequately assess corrosion using these methods, all concrete parameters must be understood [13]. Steel corrosion rate measurements, corrosion probability measurements, and electrical resistivity measurements of concrete can be obtained rapidly and reliably with electrochemical methods [14,15]. However, the current methods to evaluate the condition of corrosion in reinforcing steel do not allow an assessment of the size and a quantitative analysis of the defects. Magnetism theory can be applied to defect evaluation by comparing the magnetic properties of steel and concrete. There are several magnetic methods, including magnetic Barkhausen noise (MBN) [16], metal magnetic memory (MMM) [17], magnetic acoustic emission (MAE) [18], and magnetic flux leakage (MFL) [19,20]. Magnetic flux leakage (MFL) is the most popular magnetic sensing method used in this study to investigate defect features, including width, depth, and area. In the past, magnetic flux leakage (MFL) has been used for testing pipelines [21,22,23,24,25] and inspecting plates [26,27], tubes [28,29,30], wire ropes [31,32,33,34,35], rail tracks [36], tank floors [37,38], and suspension bridge stay cables [39] made up of ferromagnetic material.

Several magnetic studies have been conducted on defect assessment of reinforcing steel and prestressed steel tendons (see Table 1).

The prior studies are mostly related to the defect detection and region identification of corroded areas in reinforcing steel. However, few studies have explored how to quantify the cross-sectional area loss in reinforcing steel due to artificial corrosion in laboratory experiments. The geometry of defects in reinforcing steel is not well understood due to a lack of available previous research based on quantitative analyses of MFL signal components (axial, radial, and tangential). It is also a particularly challenging aspect of MFL testing when applied to reinforced concrete corrosion assessment.

The primary objective of this research was to conduct a preliminary study on defect size analysis using two-dimensional magnetic flux leakage signals (B_x_, B_y_). For this purpose, the study was designed to accomplish three main objectives: (1) to develop a numerical simulation, (2) to validate this through experimental study, and (3) to offer guidelines for the use of MFL for condition assessment of rebars. To evaluate the dynamics of magnetic flux leakage signals inside a defect and to characterize defect topology, it is crucial to identify, comprehend, and explain the contributors of the various MFL signal components.

## 2. Development of Numerical Simulation Model

### 2.1. Numerical Simulation Model

**Figure 1:**
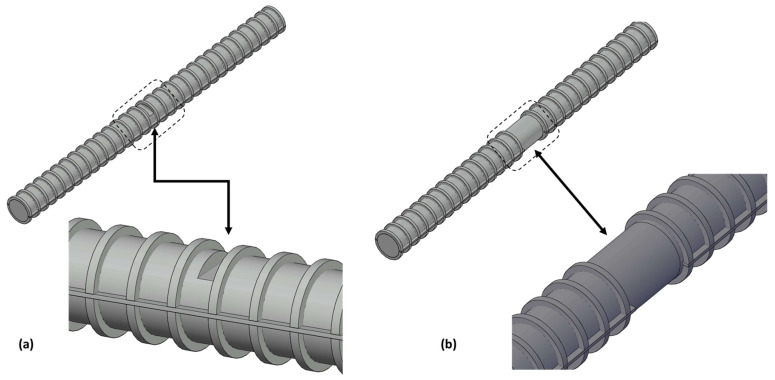
Defect types: (**a**) cutting defect, (**b**) grinding defect.

Several numerical simulations were conducted to investigate the impact of defects on two magnetic field components (B_x_, B_y_). Table 2 summarizes the defect size variables of the numerical model, also illustrated in Figure 1.

This study considered 2 types of defects: cutting defects with a consistent width and depth of 2 mm, 5 mm, and 10 mm, and grinding defects, which had 3 cases with 1, 3, and 5 rib losses. A CNC machine was used to fabricate the saw cutting defects to evaluate the consistent width and depth effect on both components of magnetic flux leakage (B_x_, B_y_). The grinding defect example was derived from localized corrosion of reinforcing steel which lost its ribs on its cross section.

**Figure 2:**
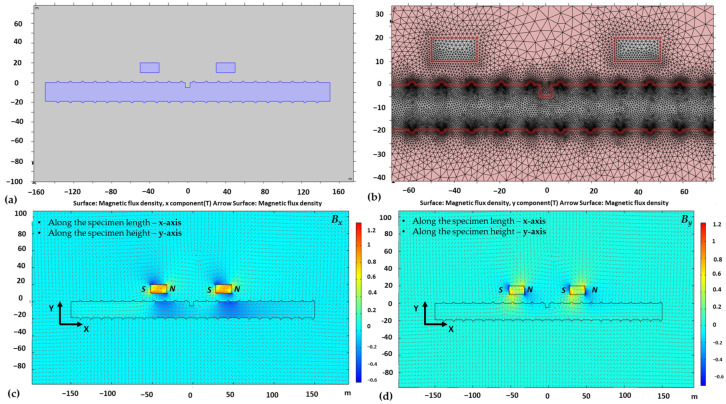
The 2D finite element model (FEM) for simulation of magnetic flux leakage components (B_x_, B_y_) on defect size of 5 × 5 in D_19_ reinforcing steel: (**a**) 2D model, (**b**) triangular meshing, (**c**) simulated FEM of x-component (B_x_) of magnetic flux leakage, and (**d**) simulated FEM of y-component (B_y_) of magnetic flux leakage.

Simulations in COMSOL Multiphysics were conducted to validate the experimental test results. By utilizing COMSOL Multiphysics, 2D modeling was simulated with different defect types using permanent magnets, resulting in magnetic flux leakage at every millimeter. Studies were carried out in an AC/DC module of COMSOL Multiphysics v5.5. Figure 2 shows the mesh analysis and results of the magnetic flux leakage components (B_x_, B_y_) on a single defect size of 5 × 5 (width × depth). The magnetization model chosen to analyze the magnetic flux leakage at the defect location is as follows:

B = µ_0_ (H + M) (1)

where B represents the magnetic flux density, H represents the magnetic field strength of the magnets, M represents the magnetization field induced in the specimen, and µ_0_ is the relative permeability of the materials. The theory states that when a magnetic field strength (H) is applied to a ferromagnetic material, magnetization fields (M) are generated within the specimen, causing leakage at the location of the defects due to differences in the materials’ permeability (µ_0_). The numerical model includes a magnetic field, no current (mfnc) as the selected physics domain. There were 3 types of geometries that were created in the 2D model, including air space, reinforcing steel, and permanent magnets. The magnet dimensions were 10 mm × 20 mm, and the magnet distance was 80 mm. As soon as the geometry was completed, the next step was to assign the material properties. The air had a permeability of 1, reinforcing steel had a permeability of 100, and the magnets had an iron permeability of 4000. To develop a permanent magnet in the COMSOL model, an iron material with the desired magnetization strength must be created. Mesh development is an essential step in achieving accurate simulation results. For this reason, custom mesh sizes were employed to reinforce the steel specimens, extremely fine mesh sizes were utilized for magnets, and finer mesh sizes were used for air space. Following a successful simulation in COMSOL, data were collected through a domain probe point option available in COMSOL for 35 mm points on either side of the defect center and at 5 mm above the specimen surface. The magnetization model included assigning the value of the magnetic field strength, taken as 860,000 A/m of Neodymium N35 grade.

#### 2.1.1. Optimization of Magnet Distance

**Figure 3:**
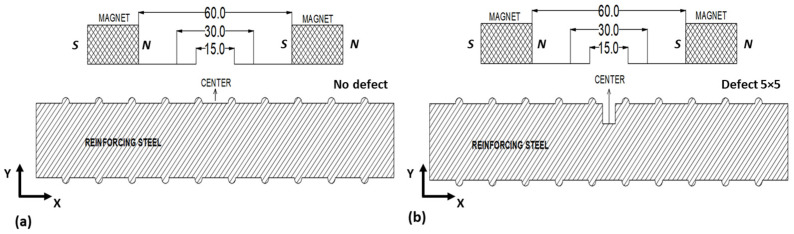
The 2D model sketches for magnet distance of specimens with (**a**) no defect and (**b**) 5 × 5 defect.

In order to obtain the maximum flux corresponding to the defect geometry, two aspects of the numerical model are crucial: the distance between the magnets and the orientation of the magnets. We studied 3 magnet distances (15 mm, 30 mm, and 60 mm) for optimizing the magnet distance for potential flux against defects with new and defected rebar specimens (see Figure 3).

**Figure 4:**
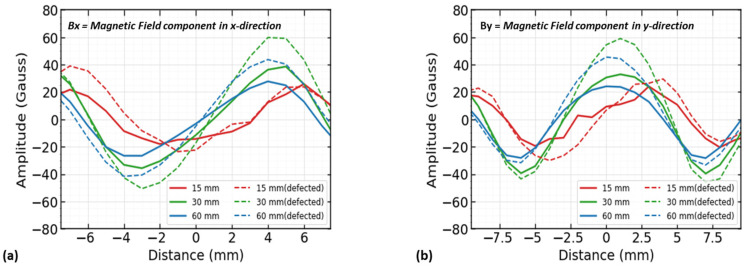
The 2D MFL signals for magnet distance: (**a**) signals of x-component of magnetic flux leakage for distances of 15 mm, 30 mm, and 60 mm, and (**b**) signals of y-component of magnetic flux leakage for distances of 15 mm, 30 mm, and 60 mm.

As part of the COMSOL Multiphysics model, data were collected using a probe located at the magnet’s center. In this analysis, our considerations included a rebar with no defect and a rebar with a defect of 5 × 5 in dimension. The analysis included the signal amplitude (peak to peak) in the model. The resulting components of magnetic flux leakage signals are shown in Figure 4.

**Figure 5:**
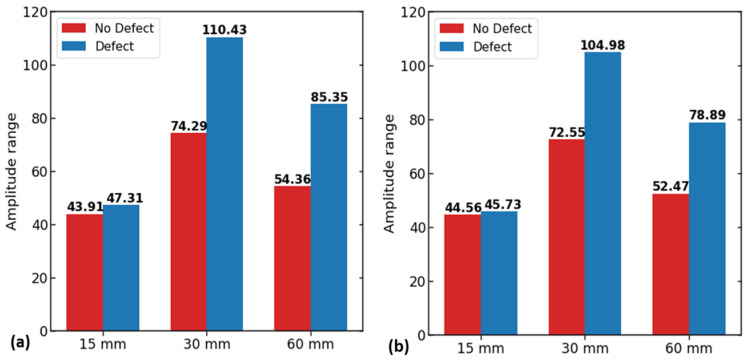
Optimum magnet distance evaluation by amplitude of magnetic flux leakage components (B_x_, B_y_): (**a**) bar plot of amplitude values of x-component; (**b**) bar plot of amplitude values of y-component.

The solid lines represent signals over the new reinforcing steel condition in Figure 4a,b, while the dashed lines represent signals over the defect. The x- and y-component signals are analyzed by the effect over their amplitude (peak to peak). A defect’s location has coordinates (0,0), and the maximum magnetic flux is at 30 mm (see Figure 5a,b). Due to the strong magnetic field between the north and south poles, the 15 mm data are disturbed and have a rough appearance. This is due to the inability of the flux to reach the probe location. Similar results can also be observed for the y-component. The distance of 30 mm between the magnets proved to be the most effective in terms of observing the signals. However, at a 30 mm distance, the magnets had such a strong magnetic field that their poles were attached to each other in practice. Therefore, separate magnets were placed on each magnet chamber in the prototype using a 60 mm distance, which also showed strong magnetic flux.

#### 2.1.2. Optimization of Magnet Orientation

**Figure 6:**
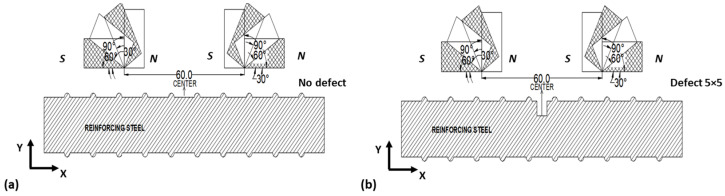
Two-dimensional model sketches for magnet orientation. Specimen with (**a**) no defect and (**b**) 5 × 5 defect.

To ensure accurate signals, magnetic flux leakage can also be affected by magnet orientation. Thus, 4 different angle orientations were analyzed in the COMSOL model: 0°, 30°, 60°, and 90°, including a rebar with no defect and a rebar with a defect of 5 × 5 in dimension (see Figure 6).

**Figure 7:**
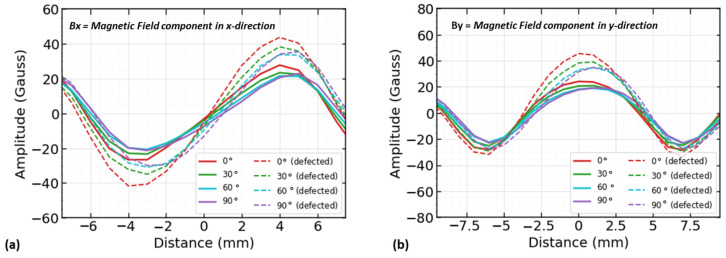
Two-dimensional MFL components for magnet orientation. (**a**) Signals of x-component of magnetic flux leakage for 0°, 30°, 60°, and 90° angle, and (**b**) signals of y-component of magnetic flux leakage for 0°, 30°, 60°, and 90° angle.

The results show that the magnet orientation at 0 degrees gives the maximum values for interpreting magnetic flux leakage signals. The solid lines represent signals over the new reinforcing steel condition in Figure 7a,b, while the dashed lines represent signals over the defect.

**Figure 8:**
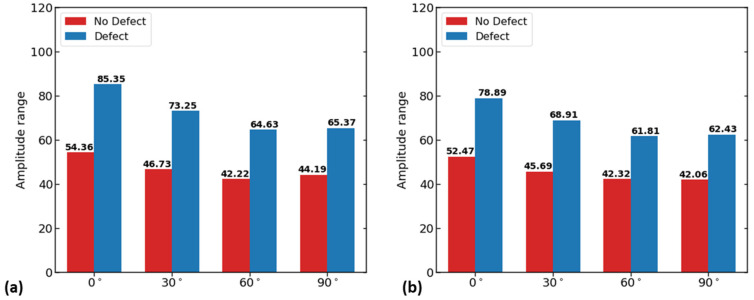
Optimum magnet orientation analysis by amplitude of magnetic flux leakage components (B_x_, B_y_). (**a**) Bar plot of amplitude values of x-component; (**b**) bar plot of amplitude values of y-component.

The results show that the magnetic flux leakage value decreases as the magnet orientation angle increases from 0° to 90°. Based on this analysis, the 0° position was selected for the magnets which aligned with the rebar surface in both the experimental and numerical models. When there is no defect or there is a defect, the highest flux is received at the 0° orientation, and at 90° the minimum flux is received, while the middle values are found in the 30° and 60° orientations (see Figure 8). Based on the above analysis for optimizing the magnet distance and orientation, a 60 mm distance and 0-degree magnet orientation were used.

### 2.2. Experimental Study

#### 2.2.1. Preparation of Test Specimens

**Figure 9:**
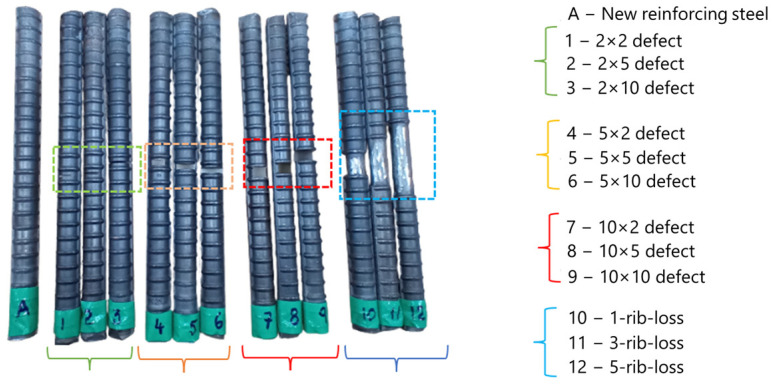
Experimental test specimens: new rebar (A), saw cutting defects (1 to 9), and grinding defects (10 to 12).

The D19-size rebar, which is typically found in reinforced concrete structures, was selected for the MFL signals in this study. For the experimental work, two types of defects were considered. The first defect type was made by using CNC machine tools to perform saw cutting, and the second defect type was made by grinding the ribs of reinforcing steel. In this study, 12 defect cases and 1 new rebar were analyzed, as shown in Figure 9.

#### 2.2.2. MFL Test Setup

**Figure 10:**
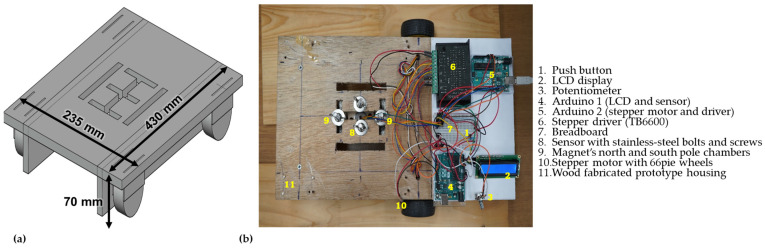
Experimental test setup: (**a**) 3D-designed model; (**b**) top view of fabricated test setup.

Several non-magnetizing materials were used to construct the experimental test prototype. It consisted of designing a housing, magnetizing the specimen with magnets, detecting magnetic field leaks using single- and triple-axis sensors, programming the Arduino UNO, displaying real-time signal data on an LCD, controlling and monitoring the scanning velocity with a stepper motor and driver, adjusting speed with a potentiometer, regulating direction with a push button, and connecting circuits on a breadboard as shown in Figure 10.

The prototype dimensions were 430 mm (L) × 245 mm (W) × 70 mm (H). The magnet and sensor locations were adjusted using stainless-steel screws and bolts. The prototype makes use of 2 rare 66 pie wheels. Figure 10 also shows the location of the electronic components.

#### 2.2.3. Summary of Test Plan

**Figure 11:**
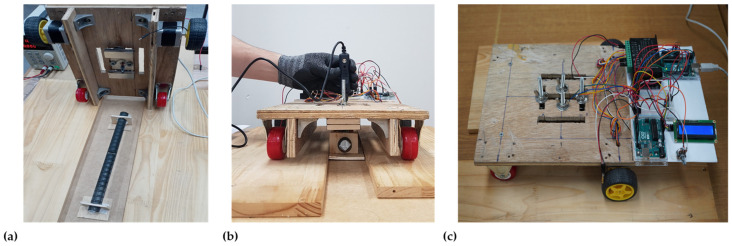
Experimental testing configuration: (**a**) bottom view with specimen, (**b**) front view during testing, and (**c**) top view of the setup.

In the experimental testing, the specimen was fixed inside a wood frame so that it could not be moved by magnetic attraction, as shown in Figure 11a. On external SerialPlot 0.10.0 software, the collected data were displayed in real time, helping to save the data from Arduino. It was found that the prototype scanning velocity impacts the optimal signals that can be analyzed when receiving signals from various defective rebars. After performing several velocity tests on the experimental setup, 10 mm/s was selected due to the stable oscillations it produced. The experimental setup also accounted for the sensor sampling frequency, which was set at 20 values per second. The simple testing configuration is shown in Figure 11.

**Figure 12:**
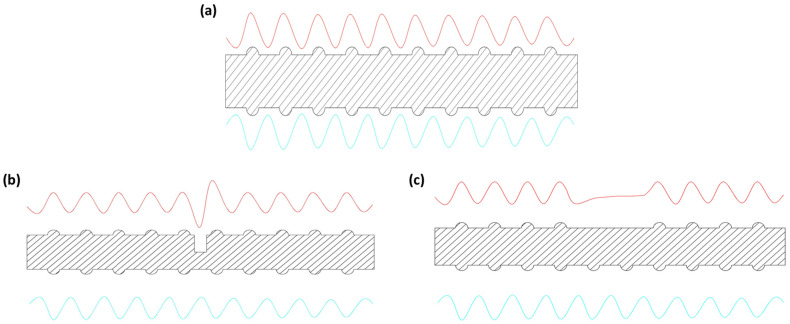
Typical waveforms of MFL signal over reinforcing steel: (**a**) new rebar, (**b**) rebar with cutting defects, and (**c**) rebar with grinding defects.

We took 3 simultaneous measurements for each of the 13 cases with each type of sensor to ensure accurate data collection. Obtaining high-quality signals is crucial for evaluating defects based on MFL readings. We examined three conditions: no defect, saw cutting defect, and grinding defect. The MFL signals exhibit a sinusoidal waveform when passing over reinforcing steel ribs, as shown in Figure 12a.

A defect in the specimen causes the signal waveforms to change in amplitude and bandwidth due to the varying defect width and depth (Figure 12b). Because of the grinding defects (rib loss), the signals no longer oscillate and are straighter, meeting the next rib peak (Figure 12c). At this point, it is suspected that there is a relationship between the MFL signal’s amplitude and bandwidth and the defect size features such as width, depth, and area.

#### 2.2.4. Sensor Positioning

**Figure 13:**
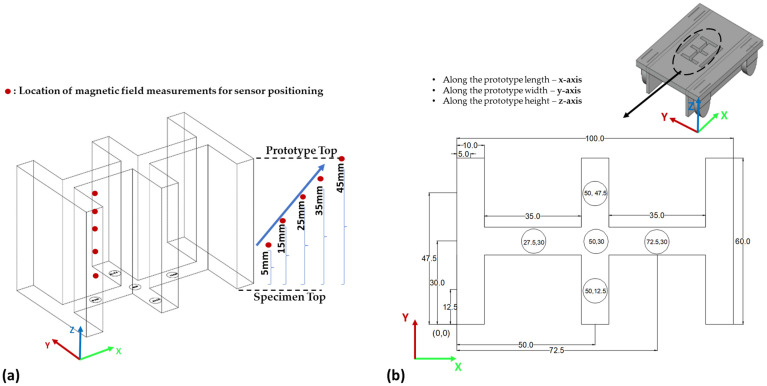
Sensor positioning coordinates in experimental prototype: (**a**) measuring point locations along the height (top of specimen to the prototype), and (**b**) coordinate measuring points in X-Y plan.

To determine the optimal location of the sensor in the test setup, magnetic field values were checked in the prototype using a Gauss meter and simple roller. Five intervals of space on the z-axis were divided, and the magnetic field was measured at each interval (see Figure 13a). There were five measurement points on the X-Y plane (see Figure 13b) at each spacing interval to ensure that the prototype had the proper sensor position. Using a Gauss meter, the magnetic field at all five points was recorded by fixing the magnetic chamber at one location. The z-direction was determined by measuring the distance from the top of the specimen to the prototype. There were 5 points in the z-direction (height): 5 mm, 15 mm, 25 mm, 35 mm, and 45 mm (see Figure 13a). The lower the value of the magnetic field in a particular location, the less the influence of the magnets in that location. Essentially, this means that the minimum magnetic value at that location will reflect the actual defect signal detected by the sensor. After recording the magnetic field at all 5 points, it was found that for the 5-millimeter height, the magnetic value was the lowest in the center position (50,30), which was 0. Consequently, the center position as the optimum location for placing the sensor was selected. As the distance from the specimen increased, the magnetic field values also increased. 

### 2.3. Validation of Numerical Simulation Model and Experimental Testing

**Figure 14:**
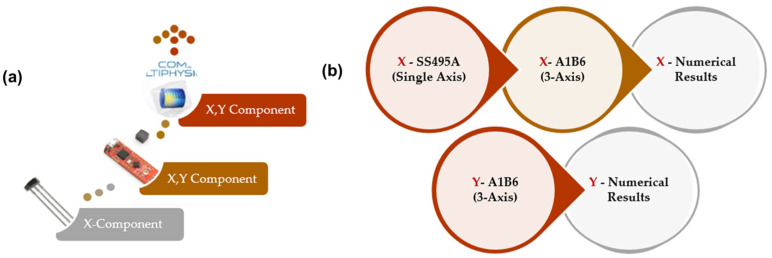
Method of cross-correlation for MFL components (X_numerical_-to-X_sensor_, Y_numerical_-to-Y_sensor_): (**a**) MFL components received from sensors and COMSOL model; (**b**) X_numerical_-to-X_sensor_, Y_numerical_-to-Y_sensor_ component cross-correlation.

The cross-correlation method was employed for studying and analyzing the similarity between the numerical and experimental signals. Cross-correlation involves analyzing the similarity of every single point of waveforms between the numerical model from COMSOL Multiphysics and the experimental signals received from the sensors. To investigate the cross-correlations, we grouped each defect signal based on its magnetic field component. In single- and triple-axis sensor signals, the x-component (B_x_) of the numerical model is cross-correlated with the x-component (B_x_) of the sensor signals. As for the y-component (B_y_), the numerical signal is cross-correlated with the equivalent triple-axis signal component. Figure 14 illustrates the cross-correlation method between x- and y-component groupings for each defect type.

**Figure 15:**
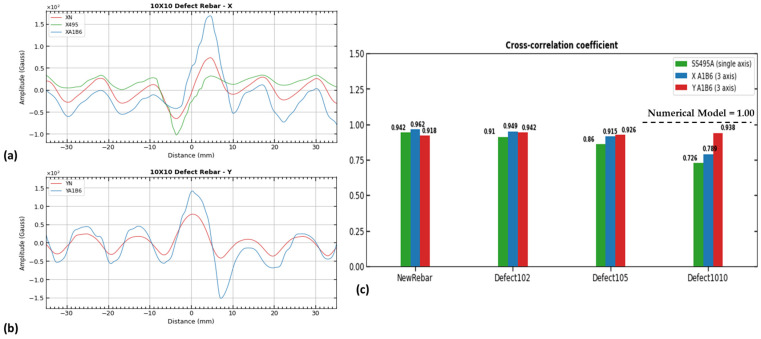
Cross-correlation of x-x and y-y component with defect size of 10 × 10: (**a**) x-component (B_x_) signals from numerical model and single- and triple-axis sensor, (**b**) y-component (B_y_) from numerical model and triple-axis sensor, and (**c**) cross-correlation coefficients bar plot for new, 10 × 2 defect, 10 × 5 defect, and 10 × 10 defect samples for experimental components (B_x_, B_y_).

An example of cross-correlation between X_numerical_-to-X_sensor_ components and Y_numerical_-to-Y_sensor_ components is shown in Figure 15. It was found that the signals from the numerical and experimental sensor components were correlated in the same way. The numerical model results and experimental work was validated using cross-correlation to study the similarity in signal waveform behavior.

Similarly, cross-correlation was applied to the grinding defect cases. The oscillation of signals disappears when the rebar ribs are missing in cases of grinding defects. While moving over the grinding surface of the reinforcing steel, a small and negligible fluctuation is observed. A change in the signal information can help interpret the rebar defect when it is detected by using the magnetic flux leakage (MFL) technique. From Table 3 below, the sensor efficiency and overall cross-correlation behavior between the experimental and numerical signals results were also evaluated. The coefficients of the cross-correlation for all 13 cases are shown in Table 3.

**Figure 16:**
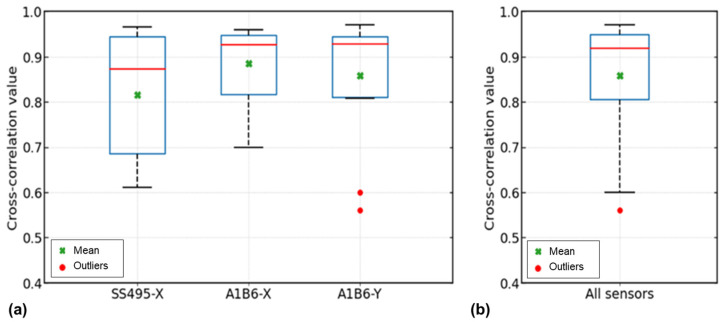
Sensor efficiency evaluation from cross-correlation coefficients: (**a**) cross-correlation of single- and triple-axis sensor components; (**b**) box plot for all cross-correlation coefficients of experiments.

We determined which sensor and its magnetic field components were closest to the numerical signal data when evaluating the sensor efficiency. As shown in Figure 16, cross-correlated data from Table 3 were plotted into a box plot to determine the mean and median values for each sensor and its magnetic field component.

As seen in Figure 16a, the 3-axis sensor (A_1_B_6_) with the x-component has the highest correlation (0.933) with the numerical signal but has a wider data distribution than the y-component. This is less spread out in the three-axis sensor (A_1_B_6_) with the y-component signal data, but two outliers are evident. With the single-axis sensor (SS495), the distribution of the cross-correlation coefficients is wider, and the median is also lower. The three-axis sensor had a better correlation with the numerical model than the single-axis sensor and demonstrated good overall performance. A box plot for all defect values was plotted to examine the correlation between the experimental sensors and the numerical model. With regards to the above box plot (see Figure 16b), the mean value for all the experimental cross-correlation data is 0.860, with a median value of 0.920. According to the median (0.920), mean (0.860), and interquartile range (0.803–0.949), the 2 sensors were accurate in capturing signals during the experiments, and therefore their signal data can be used to evaluate defects. The analysis of the 39 cross-correlation values proves that the numerical and experimental work was carried out correctly.

## 3. Parametric Study

### 3.1. Signal Variables

**Figure 17:**
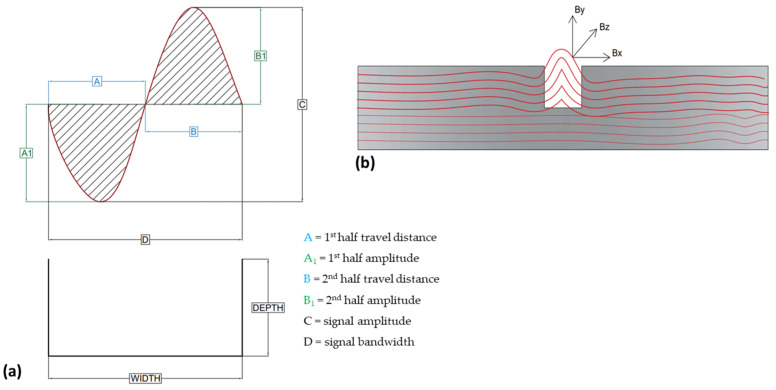
Signal study structure: (**a**) signal variables and (**b**) typical magnetic field components.

In order to study very close changes in signal features, the signals were separated into six components as shown in Figure 17a.

While studying the signal structure, it was observed that the x-component (B_x_) of the sensor correlates better with defect width as it is parallel to the defect. On the other side, the y-component (B_y_) shows good correlation with defect depth which is perpendicular to the defect. In practice, each point in space has three directions of magnetic field components, B_x_, B_y_, and B_z_, which is demonstrated in Figure 17b.

### 3.2. Results

#### 3.2.1. Numerical Simulation Results

**Figure 18:**
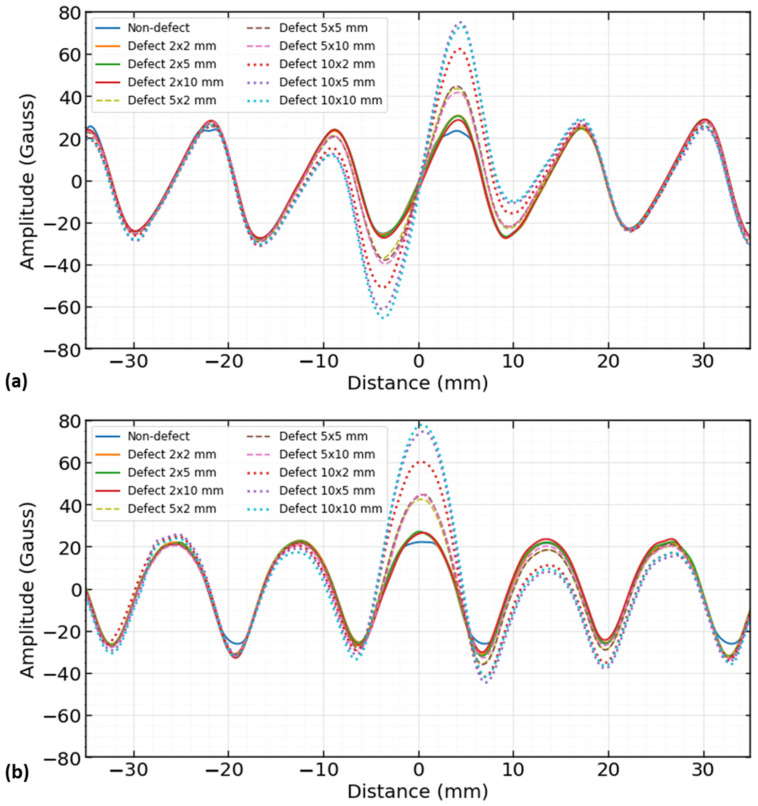
Numerical simulation model results: (**a**) MFL x-component (B_x_) and (**b**) MFL y-component (B_y_).

In COMSOL Multiphysics, a 2D finite element model (FEM) of reinforcing steel for magnetic flux leakage was developed to determine the 2 components (B_x_, B_y_) of magnetic field leakage. In the FEM simulation, the original values are presented in Tesla, which is a larger unit of the magnetic field, and then converted into the Gauss unit once all the experimental data were collected. Data were converted in one unit (Gauss), which is also the smallest unit, so that the data were as sensitive as possible. The signals from the smallest unit show greater variability, which can be useful when studying defect characteristics. In the Gauss meter data analysis, magnetic flux leakage measurements were taken every millimeter and at a height of five millimeters where good signals were observed. The results of the numerical simulation model are shown in Figure 18.

In Figure 18, it can be seen that a unique magnetic flux leakage signal was received for each type of defect size, and it is suspected that each signal has information of its corresponding defect features. The defect center is coordinated at (0,0) with a 35 mm distance on either side of the defect center. At least two ribs were considered to visualize the difference between the defected and non-defected region over the reinforcing steel.

#### 3.2.2. Experimental Testing Results

**Figure 19:**
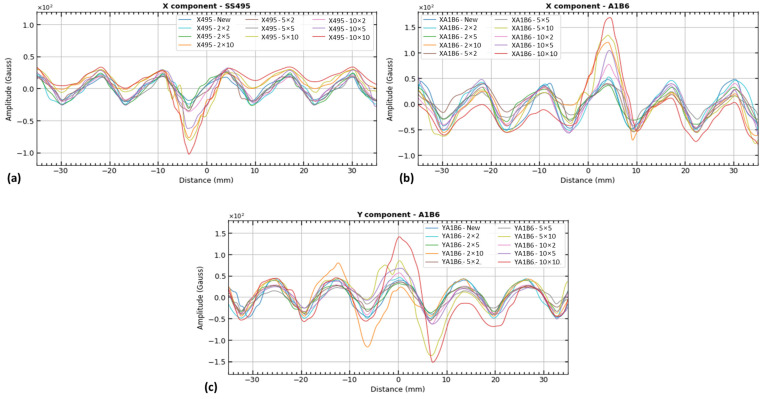
Experimental results from single- and triple-axis sensor components: (**a**) x-component of single-axis sensor (SS495A Hall effect sensor), (**b**) x-component of triple-axis sensor (TLV493D—A_1_B_6_), and (**c**) y-component of triple-axis sensor (TLV493D—A_1_B_6_).

In the experimental work, the combined variation in signal waveforms through single- and triple-axis sensors on cutting defects were studied. A trend towards increasing signal amplitude and bandwidth was observed as the defect size increased. Considering these factors, we developed the hypothesis that the signal has information about defect features such as width, depth, and area. It was also noticed that the signal waveforms showed similar characteristics both experimentally and numerically for the same magnetic flux leakage components (B_x_, B_y_). Consistent defect size changes in width and depth resulted in significant variations in output signals. Using single- and triple-axis sensors, all the signals of magnetic flux leakage over the cutting defects of varying width and depth cases were plotted as separate magnetic field leakage components (B_x_, B_y_), as shown in Figure 19.

Afterward, each case was analyzed, and the signal was processed in detail using advanced data analysis tools such as Python and MATLAB. Analyzing and comparing detailed data is imperative in understanding the impact of magnetic flux leakage.

#### 3.2.3. Effects of Defect Width and Depth

**Figure 20:**
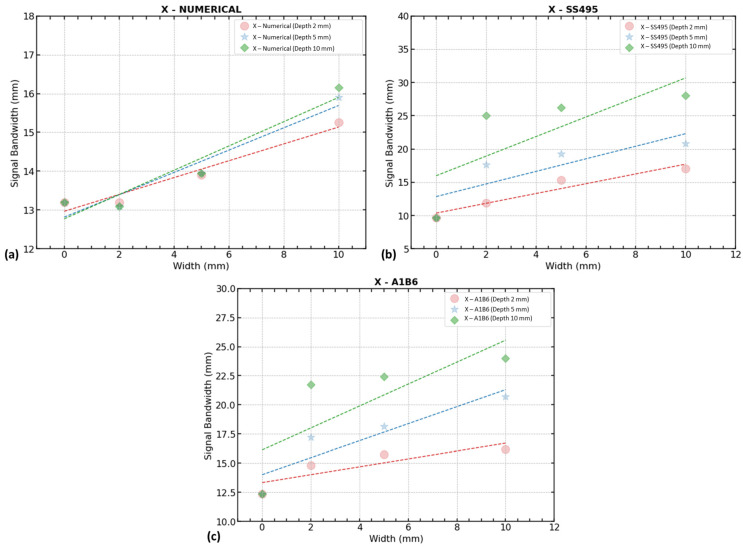
Defect width trend by signal bandwidth of x-components: (**a**) numerical model (B_x_), (**b**) single-axis sensor (B_x_), and (**c**) triple-axis sensors (B_x_).

By analyzing each signal component separately, it was determined that the trend in signal bandwidth (D) of the magnetic flux leakage x-component (B_x_) shows a correlation with defect width. In the numerical model, the defect depth shows no impact on the width data trend because the line equations are almost overlapping with each other. In the experimental work, the data show substantial changes as the defect depth increases. Both single- and triple-axis sensors show a similar pattern of an increasing trend with almost identical values of trendline slope. It is found that in the defect width evaluation there is an influence of defect depth, making it difficult to evaluate them individually. A linear plot was used to depict the three x-components from the numerical model, single-axis sensor (SS495), and triple-axis sensor (A1B6) data (see Figure 20).

**Figure 21:**
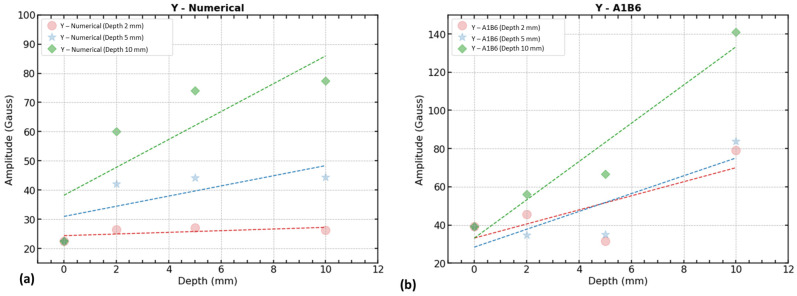
Defect depth trend by signal 1st half amplitude of y-components (B_y_): (**a**) numerical model (B_y_) and (**b**) triple-axis sensor (B_y_).

Specifically, when looking at the defect depth analysis, it was determined that the magnetic flux leakage y-component (B_y_) with the first half amplitude (Figure 17a) shows a good correlation with the defect depth. Similar to the width evaluation, linear regression plots were used to demonstrate the increasing trend of defect depth with the y-component of the magnetic field (B_y_) from the numerical model and triple-axis sensor (see Figure 21).

The numerical model revealed that defect width has a considerable impact on defect depth data trends (Figure 21a). In the experimental 3-axis sensor with the y-component (B_y_), significant changes occur in the signals when the defect is wider than 5 mm. The amplitude has a higher range from 65 to 141 compared with widths of 2 mm and 5 mm which have amplitudes ranging from 30 to 80 (Figure 21b).

#### 3.2.4. Defect Area Estimation

The signal analysis and detailed defect investigation indicate that defect width and depth cannot be evaluated separately in MFL signals since both impact each other. Therefore, signals with the same depth but varying widths (2 × 2, 5 × 2, 10 × 2) will have different signals, while those with the same width but varying depths (2 × 2, 2 × 5, 2 × 10) show different signals. However, the MFL signals can be used to estimate the defect area. The measurement process focuses on the maximum amplitude and the full bandwidth (Figure 17a) of the signal waveform at the defect location. We evaluated which sensor has the highest regression coefficient (R^2^) and identified the magnetic field components that correlated better with the defect areas. With the information in the signal features, the amplitude and bandwidth of the magnetic flux leakage component values were extracted.

**Figure 22:**
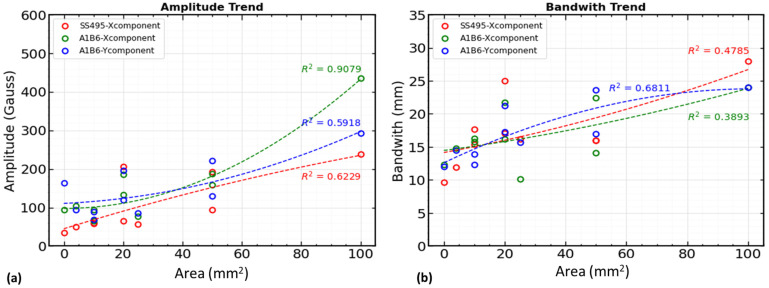
MFL signal components’ correlation with defect areas. (**a**) Amplitude correlation from single- and triple-axis sensor components, and (**b**) bandwidth correlation from single- and triple-axis sensor components.

In Figure 22, single- and triple-axis sensor component data for amplitude and bandwidth is correlated to defect areas. Figure 22a indicates that the triple-axis sensor amplitude with the x-component (B_x_) shows the highest regression coefficient (R^2^ = 0.9079) compared with the single-axis sensor (R^2^ = 0.6229) and the y-component of the triple-axis sensor (R^2^ = 0.5918). The regression coefficients for the bandwidth from two components of the triple-axis sensor (Figure 22b) are lower than those for the amplitude. This shows that the amplitude of signal waveforms from the x-component (Figure 22a) governs the characteristics of the defect areas. In other words, a triple-axis sensor with an x-component (B_x_) can be used to detect and estimate defect areas in reinforcing steel using the MFL technique. A good correlation between the triple-axis sensor signals and the numerical model data was also observed from the cross-correlation coefficients (Figure 16a). As shown in Figure 22, these are the polynomial regression coefficients for the amplitude and bandwidth trends for both sensors’ magnetic flux leakage components (B_x_, B_y_).

### 3.3. Guidelines for Condition Assessment of Reinforcing Steel Using MFL

A detailed analysis of the defect width, depth, and area in bare reinforcing steel has been carried out in this preliminary study using two-dimensional magnetic flux leakage components. Reinforcing steel is embedded in concrete that varies in thickness. A key aspect of the MFL technique is capturing the flux leakage accurately while fully saturated magnetization is achieved. The defect location can be easily determined after receiving the magnetic flux leakage signals, but defect size can only be determined after analysis of the signals received. In this study, we found that a triple-axis sensor with an x-component (B_x_) can be used to evaluate the defect area in a two-dimensional analysis. The volumetric loss of defect can be further understood by including the third component of the magnetic flux leakage (B_z_, tangential component). When the sensor is positioned in its optimum location, which we found to be in the middle of the magnet’s distance and as close to the specimen as possible, modern three-axis magnetic field sensors can evaluate defect characteristics. Signals from sensors can be affected by magnet distances and orientations. As a result of the large number of data sets varying in defect shapes, sizes, and diameters, a comprehensive prediction model of defect size analysis can be developed to correlate magnetic flux leakage signal loss to defect volumetric mass loss. As a commercially ready-to-implement MFL testing apparatus for localized corrosion detection is not available yet, this study provides strong evidence for the design, development, and testing of a model of a modern flux leakage device inside a research facility. This can help for the condition assessment of reinforcing steel in concrete structures from an infrastructure management agency’s perspective.

## 4. Conclusions

This experimental and numerical study on the detection and characterization of defects in reinforcing steel by using magnetic flux leakage components has led us to draw the following conclusions:

1. MFL signals exhibit a sinusoidal waveform over the ribs of reinforcing steel. The cross-correlation between the experimental sensor signals and numerical model results is high. Compared with a single-axis sensor, the 3-axis sensor presents closer values to the numerical model data as the x-component has the highest cross-correlation coefficient median (0.933) and mean (0.860).
2. In the investigation using signal information for defect prediction, the bandwidth of the x-component of the signal (B_x_) correlates with defect width. In the case of increasing depth, it corresponds to the first half amplitude (A_1_) of the y-component (B_y_).
3. In this 2D MFL signal study, both parameters of the 2D defect (width and depth) affect each other and cannot be evaluated individually. The defect area can be estimated from the overall variation in the signal amplitude of the magnetic flux leakage signals with an x-component (B_x_). The defect areas show a higher regression coefficient (R^2^ = 0.9079) for the x-component (B_x_) amplitude of the 3-axis sensor signal.

This study shows consistent experimental and simulation results, which demonstrates that MFL is an efficient method for evaluating the condition of reinforcing steel. One of the major limitations of this technique is the signal processing and analysis of oscillated waveforms caused by reinforcing steel ribs. Moreover, a limited number of defect sizes have been considered in this preliminary study, whereas developing a comprehensive defect prediction model for rebars requires a large number of data samples. Developing more accurate interpretation approaches can be facilitated by advanced laboratory work. Future studies will focus on examining in more depth the quantitative relationship between measurements of magnetic flux leakage and cross-sectional area loss in reinforcing steel. In addition to investigating all three components of magnetic flux leakage (axial, radial, and tangential), research will also be conducted on the volumetric mass loss of reinforcing steel caused by localized corrosion. This needs to be further studied to make it fully functional in conjunction with future IoT applications. The results presented lay the foundation for the creation of corrosion detection and quantification methods based on the MFL principle for embedded reinforcing steel under various concrete cover thicknesses.

## Figures and Tables

**Table 1:** Summary of magnetic studies on defect evaluation in reinforcing steel and steel tendons.

Researchers	Location	Target	Experimental Scale		Defect Characterization		Remarks
			Laboratory	Actual Structure	Detection	Quantification	
Shams et al. [40]	USA	Steel Tendons	√	√	√	X	Corrosion extent was identified on prestressed steel strands using magnetostatic and transient numerical method
Q. Zhao et al. [41]	China	Reinforcing Steel	√	X	√	X	For evaluating steel bars’ rust, the linear rate of change in the tangential component curve of magnetic flux leakage signals was used
D. Yang et al. [42]	China	Reinforcing Steel	√	X	√	X	According to the magnetic dipole model, self-magnetic flux leakage for V-shaped corrosion defects was simulated, and an experimental index “K” was established to characterize corrosion severity for steel bars
H. Scheel et al. [43]	Germany	Steel Tendons	X	√	√	X	Magnetic field sensor signals of tendons were used for detecting prestressed wire fractures in pre-tensioned and post-tensioned concrete structures
B. Fernandes et al. [44]	USA	Steel Tendons	X	√	√	X	With the magnetic flux leakage method, hidden corrosion and prestressing strand breaks of box-beam bridges were predicted
M. Mosharafi et al. [45]	Canada	Reinforcing Steel	√	X	√	X	A magnetic-based method was used to assess the magnetic properties of defective rebars with transverse cracks
H. Zhang et al. [46]	China	Reinforcing Steel	√	X	√	X	Three-dimensional digital micromagnetic-sensor-based measurements for tangential curves of magnetic field were used to evaluate and detect inner steel corrosion by self-magnetic flux leakage
H. Azari et al. [47]	USA	Steel Tendons	√	X	√	X	To detect cross-sectional loss in steel strands in prestressed concrete bridge girders (I-beams), a dynamic test setup was used to inspect the corroded zone
H. Diederich et al. [48]	Switzerland	Reinforcing Steel	√	X	√	X	By developing a simultaneous magnetization and defect detection device, a qualitative analysis of magnetic flux leakage signals was conducted under various defect conditions of reinforcing steel
M. Mosharafi et al. [49]	Canada	Reinforcing Steel	X	√	√	√	The iCAMM scanner device was used to assess the corrosion of reinforcements in a bridge deck using magnetic methods
Y. Sun et al. [50]	China	Reinforcing Steel	√	X	√	X	By introducing an open electromagnetic coil excitation technique, defects in rebars were detected by magnetic flux leakage
C. Fu, J. Huang, Z. Dong et al. [51]	China	Reinforcing Steel	√	X	√	√	An electromagnetic-based apparatus was developed for detecting and monitoring corrosion in reinforcing steel, and a linear relationship was found between the magnetic sensor signals and the corrosion mass loss
S. Jiang et al. [52]	China	Reinforcing Steel	√	X	√	√	The cross-sectional area and weight loss ratio for rebar in uniform corrosion conditions were calculated using partial modulus and magnetic gradient, which consider the rebar’s own magnetic characteristics
J. Qiu et al. [53]	China	Reinforcing Steel	√	X	√	√	By utilizing different quantitative indicators, a quantitative evaluation of a cross-sectional area of rebar was proposed based on the self-magnetic flux leakage method in localized corrosion
Z. Li et al. [54]	China	Reinforcing Steel	√	X	√	X	Self-built electromagnetic apparatus equipped with Hall effect sensors was used to monitor and detect corrosion of reinforcing steel in concrete

√: applies; X: does not apply.

**Table 2:** Main variables of the numerical simulations in this study (D_19_).

Simulation Case		Dimension of Defects	
		Width (mm)	Depth (mm)
New Rebar	Specimen A	0	0
Saw Cutting Defect (CNC Machine)	Specimen 1	2	2
	Specimen 2	2	5
	Specimen 3	2	10
	Specimen 4	5	2
	Specimen 5	5	5
	Specimen 6	5	10
	Specimen 7	10	2
	Specimen 8	10	5
	Specimen 9	10	10
Grinding Defects (Rib Loss)	Specimen 10	1 Rib Loss	
	Specimen 11	3 Rib Loss	
	Specimen 12	5 Rib Loss	

**Table 3:** All defects’ cross-correlation coefficient results.

Experimental and Numerical Cross-Correlation												
New	Saw Cutting Defects									Grinding Defects		
	2 × 2	2 × 5	2 × 10	5 × 2	5 × 5	5 × 10	10 × 2	10 × 5	10 × 10	1 Rib	3 Rib	5 Rib
**X-Component—SS495 Hall Effect, (Single-axis Sensor) Correlation**												
0.942	0.951	0.949	0.656	0.941	0.966	0.694	0.91	0.86	0.726	0.886	0.65	0.611
**X-Component—TLV493D-A1B6, (3-axis Sensor) Correlation**												
0.962	0.959	0.947	0.70	0.821	0.933	0.803	0.949	0.915	0.789	0.957	0.92	0.939
**Y-Component—TLV493D-A1B6, (3-axis Sensor) Correlation**												
0.918	0.971	0.970	0.808	0.951	0.892	0.6	0.942	0.926	0.938	0.93	0.81	0.56

## Data Availability

The article contains data. However, the corresponding author can provide you with the data presented in this study upon request.
